# Twenty-ninth Annual Commencement Exercises

**Published:** 1872-01

**Authors:** 


### Chicago Medical Journal.

A MONTHLY RECORD OF

*Medicine, Surgery and the Collateral Sciences.*

EDITED BY J. ADAMS ALLEN, M.D., LL.D.; AND WALTER HAY, M.D.

VOL. XXIX.—JANUARY, 1872.—NO. I.

### RUSH MEDICAL COLLEGE — *Twenty-ninth Annual Commencement Exercises.*

The Rush Medical College had the exercises of its Twenty-ninth Annual Commencement, Wednesday evening, Jan. 17th, in the east lecture room of the Michigan Avenue Baptist Church. The place was crowded with students and friends of the institution. The diplomas were delivered to the graduating class with the usual formula. There were seventy-seven graduates. They were a good, honest, intellectual looking set of men, and will do credit to their Alma Mater. The oldest student in the class is thirty-seven years of age, the youngest twenty-one. The average is about twenty-five. The names of the graduates follow:

LIST OF GRADUATES.

| *Name.* | *Residence.* |
|---|---|
| O. J. H. ADAMS, | Chicago, Illinois. |
| WM. F. ARTZ, | Byron, Illinois. |
| CHAS. H. BURBANK, | Campton, Kane county, Illinois. |
| CHAS. E. BOOTH, | Elroy, Juneau county, Wisconsin. |
| E. C. BARTHOLOW, | Mahomet, Illinois. |
| S. S. CLAYBERG, | Fairview, Fulton county, Illinois. |

| | |
|---|---|
| ALBERT CHENOWETH, - - | Decatur, Illinois. |
| F. ANTES CANFIELD, - - | Necedah, Wisconsin. |
| H. S. CHAPIN, - - - - | Dewitt, Illinois. |
| THOS. N. CUNNINGHAM, - - | Havana, Illinois. |
| CHAS. T. CORY, - - - - | Patch Grove, Grant county, Illinois. |
| O. P. CRANE, - - - - | Mason City, Illinois. |
| J. C. DORCHESTER, - - - | Chicago, Illinois. |
| L. H. DUNNING, - - - | Edwardsburg, Michigan. |
| T. B. DEWITT, - - - - | Milan, Sullivan county, Missouri. |
| J. W. DUNN, - - - - | Lamar, Barton county, Missouri. |
| D. B. DARR, - - - - | Ladora, Iowa county, Iowa. |
| C. M. EASTON, - - - | Norton, Kankakee county, Illinois. |
| W. W. EDGERTON, - - - | Sparta, Wisconsin. |
| J. McL. FLEMING, - - - | Chicago, Illinois. |
| E. W. FAIRMAN, - - - - | Oxfordville, Rock county, Wisconsin. |
| S. C. FREELAND, - - - | Portersville, Dubois county, Indiana. |
| JOHN H. GARDINER, - - - | Mahomet, Illinois. |
| JOHN H. GERNON, - - - | Lafayette, Indiana. |
| E. S. GARVIN, - - - - | Sycamore, Illinois. |
| JOHN GARDNER, - - - | Davisville, Yolo county, California. |
| G. B. GALER, - - - - | Dewitt, Clinton county, Iowa. |
| R. H. HUDDLESTON, - - | Farmer City, Illinois. |
| P. S. HAYES, - - - - | Chicago, Illinois. |
| R. S. HALL, - - - - | Waterloo, Iowa. |
| W. H. HILL, - - - - | Dayton, Tippecanoe county, Indiana. |
| W. F. HILSABECK, - - - | Windsor, Illinois. |
| S. M. JENKS, - - - - | Cokato, Minnesota. |
| E. J. KENDALL, - - - | Waupecong, Indiana. |
| G. W. LASHER, - - - - | Kane, Greene county, Illinois. |
| W. K. MILLER, - - - - | Batavia, Jefferson county, Iowa. |
| W. L. McCANDLESS, - - - | Pinckneyville, Illinois. |
| OTIS MOORE, - - - - | Chicago, Illinois. |
| L. C. MESSNER, - - - - | Marysville, Illinois. |
| HORATIO N. MACKEY, - - | Morganstown, West Virginia. |
| R. L. McKINNIE, - - - - | Viola, Illinois. |
| ANDREW McFARLAND, - - | Jacksonville, Illinois. |
| S. K. McBRIDE, - - - - | New Alexandria, Pennsylvania. |
| W. B. MEAD, - - - - | Huntsville, Illinois. |
| P. H. MILLARD, - - - - | Lisbon Centre, New York. |
| D. E. MAGOON, - - - | Whitewater, Wisconsin. |
| A. A. VON MANSFELDE, - - | Chicago, Illinois. |
| M. C. McPHERSON, - - - | Eagle Point, Ogle county, Illinois. |
| G. F. MERRITT, - - - - | Burlington, Iowa. |
| J. W. NORRIS, - - - - | Mackinaw, Illinois. |
| A. L. NORRIS, - - - - | Farmer City, Illinois. |
| A. OWEN, - - - - | Texana, Jackson county, Texas. |
| C. W. PHILLIPS, - - - - | Springfield, Illinois. |
| H. H. PRATT, - - - - | Crown Point, Indiana. |
| Z. E. PATRICK, - - - - | Washington, Illinois. |
| H. J. PRATT, - - - - | Montello, Michigan. |
| B. PLACKETT, - - - - | Wanakee, Dane county, Wisconsin. |
| D. L. RUSSELL, - - - | Versailles, Illinois. |
| D. L. ROSS, - - - - - | Chicago, Illinois. |
| A. N. RICHARDSON, - - | LaMoille, Illinois. |
| G. F. ROBERTS, - - - - | Metamora, Illinois. |
| A. B. STRONG, - - - - | Gambier, Ohio. |

| | |
|---|---|
| J. I. SMITH, - - - - - | Lena, Stephenson county, Illinois. |
| W. F. STANDIFORD, - - - | Westville, Indiana. |
| E. J. SMITH, - - - - | Cedar Rapids, Iowa. |
| CYRUS SMITH, - - - - | Anoka, Minnesota. |
| J. W. STANDLEY, - - - - | Kirksville, Adair county, Missouri. |
| O. P. SMITH, - - - - | Corydon, Wayne county, Iowa. |
| J. A. SMITH, - - - - | Cedar Rapids, Iowa. |
| S. C. THOMPSON, - - - | Merton, Waukesha county, Wisconsin. |
| E. K. McA. TAYLOR, - - - | Le Roy, McLean county, Illinois. |
| GEO. H. TEBO, - - - - | Mount Sterling, Brown county, Illinois. |
| J. G. TRUAX, - - - - | Fort Atkinson, Wisconsin. |
| THOS. L. A. VALIQUET, - - | Chicago, Illinois. |
| R. W. WELLS, - - - - | Texana, Jackson county, Texas. |
| S. J. WAY, - - - - - | Kankakee City, Illinois. |
| O. B. WIGGINS, - - - - | Lawrence, Michigan. |
| *Ad Eundem—* | |
| ERNST SMITH, - - - | Chicago, Illinois. |

### CHARGE TO THE CLASS.

Dr. J. W. Freer, President of the institution, delivered a brief charge to the class, which contained much sensible advice. He said :

Gentlemen of this graduating class: The highest honors of this institution, by which you are initiated into the medical fraternity, have now been conferred upon you. The testimonials which you bear away will be respected by the intelligent public as so many assurances of your learning, skill, and scientific attainments. Your future conduct must determine how well you have deserved these tokens at the hands of your *Alma Mater*. It is customary on such occasions as this to address the young alumni with friendly words of advice and admonition, with the hope and expectation that their future conduct as physicians and citizens may thereby be influenced for good. It is also a time-honored custom to expatiate largely upon the sacredness of our calling. We have no objections to this when appropriate and opportune. But in this instance, knowing as we do, by personal intercourse, the superior degree of intelligence and high moral tone of the present graduating class, we deem it quite unnecessary to try their patience with a long list of the benefits to be derived from the practice of virtue, temperance, benevolence and industry.

We have frequently during the session offered such advice as we deemed useful, and particularly have we advised that you should make up your minds to devote all your leisure moments, during your whole natural lives, to the pursuit of this study of science that you have as yet but skimmed over in the most superficial manner. For these purposes the early part of your career as physicians will, I might say fortunately for many, afford you many spare days. You have now learned how to learn, and how to study, and this is a great advantage, and the first thing in importance in the acquirement of knowledge. We have shown you the proper methods of study, of the functions of different kinds of living and dead animals, by experiment and otherwise, by which you may become familiar with the physiological phenomena before attempting to unravel the pathological conditions. Repeat these experiments and observations until the details are engraven upon the tablet of your memories. Make every reasonable sacrifice in order to gain possession of microscopes that you may continue your studies.

The necessity of continued study was further inculcated in energetic and appropriate terms. The essentials to the success of the physician were named as follows: "A gentlemanly exterior, a cultivated address, a neat, commodious, well-furnished office, a large, well-selected library, and all necessary instruments and apparatus." The obtaining of these essentials would occupy fully the time of the young physicians before the speaker, and save the necessity of lounging in the corner grocery. The characteristics of the unsuccessful physician were next enumerated. They were a lack of neatness, as shown by a cob-webbed office, and carelessness in providing the tools essential to the prosecution of his business. The address closed as follows:

In conclusion, gentlemen, allow me, in behalf of the Trustees and Faculty of Rush Medical College, to express to you our sense of obligation for the noble manner in which you have sustained us during our days of trial. You enjoyed our beautiful college edifice but a few days; you saw it sink in ashes, but stood by like friends in need. At that time the world's great heart palpitated with sympathy for the dreadful losses sustained by your *Alma Mater*. The doors of the most renowned schools, as well as those of lesser note, were thrown wide open to you free of charge, wherein you might finish your course of lectures. But you declined these generous offers, and chose to remain with the school of your first choice, notwithstanding circumstances had driven it to humbler halls and plainer surroundings. The result of our course has proven that ornate walls of brick and mortar are not essentials of successful medical teaching, for no preceding class has given greater satisfaction at the final examination than that of 1871 and 1872.

### CLASS VALEDICTORY.

The class valedictory was delivered by O. J. H. Adams, as follows:

*Mr. President, and Gentlemen of the Faculty of Rush Medical College:*

The twenty-ninth graduating class of this institution will now, through me, their representative, respond to the charge just given.

To the thinking mind, in all ages of the world, three important questions have ever been presented, viz: What am I? Whence my origin? What my destiny? The answers to these three questions came in process of time from two sources. First, to man from his Creator; secondly, from man himself. The first known as revelation; the second, reason—so called.

When man first asked these questions, and a reply came from his Creator, he was doubtless content; but time passed—generation after generation arose; this reply was given from father to son. Some accepted it, and were satisfied; others, the greater number, wise above their day and generation, added to or detracted from, as their reason, so called, dictated; and here arose the contest between reason and revelation, the breach widening from that until the present.

As the reply from the Creator—revelation—has never changed, and is familiar to you all, we will not repeat it here, but proceed to notice the replies given by man.

The replies given by man, the offspring of reason, have continually changed as time has changed.

We shall only notice the two recorded extremes, as presented near three thousand years ago, and at the present time. Near three thousand years ago, when a philosopher turned his thoughts within and asked himself these questions, there came the following replies:

What am I? A god or a demi-god.

What the source of this intelligence I manifest? The divinity within.

Whence my origin? From the gods.

How? In accordance with the divine law of procreation.

What my destiny? Immortality.

Here we find part of the fundamental principles presented in revelation, but so modified as to be more acceptable to man's pride.

At the present time, after the passage of those thousands of years, during which human reason has been continually at work—reason independent of everything except its own inherent powers—we receive the following replies from the same class of men to the same questions.

What am I? A baboon.

What the source of this intelligence I manifest—this reason? The brain and nervous system, acting on the principle of a galvanic battery.

Whence my origin? From a species of porifera.

How? By mere chance.

What my destiny? Annihilation.

Human reason continuously employed through thousands of years upon one topic, man; commencing in the belief of the divinity and immortality of man, and the result summed up runs thus:

I now am a baboon.

My mental manifestations are the results of magnetism.

My parents millions of years ago were a species of porifera. They came into existence by mere chance.

In death I shall be annihilated.

When in early youth we were first permitted to indulge in perusing the classic lore of Greece and Rome, and in our imaginations took our stand on Ilium's walls, and there beheld men, gods, and demi-gods, all engaged in one bloody conflict, whilst the Eternal, seated on snow-capped Olympus, from his mighty arm hurled athwart the murky sky his flaming bolts, we were entranced. Or from Mount Ida's top, at early morn, we beheld Aurora seated in her golden chariot, with foaming steeds, dashing through the eastern sky to unbar the gates of light, we were charmed.

This theology of three thousand years ago, as modified by man, possessed a peculiar fascination in flattering man's pride, and feeding his imagination, that rendered its adoption somewhat excusable; but this theology, if we may so speak, *the offspring of reason alone*, received by the same class of men in modern times, is certainly devoid of one single charm to recommend it to any one. Could it possibly be true, its acceptance must necessarily commence in humiliation, run into degradation, and finally end in ruin. The belief in death being an endless sleep is enough of itself to produce all this.

Revelation, on the other hand, when truly accepted, commences immediately to elevate morally, and render life more desirable; and as time passes, it continues to elevate morally, and increase one's happiness; and when the hour arrives for the great change known as death, it disarms the grave of all its terrors, and enables the departing one to meet death joyfully. This assertion has been verified in the life and death of millions.

If now it were possible for revelation to prove untrue, and there be no hereafter, it certainly has done the deceased one no injury—his endless sleep will be just as sweet as though he had died a devoted disciple of modern theology. But, on the other hand, should it prove true—which it certainly will—what is there in the possession of man that he would not give, or in the power of man that he would not do, that in death he might be enabled to realize such a truth?

A selection between the theology of man and that of revelation is not difficult. WE unhesitatingly take the side of revelation.

Entertaining this view concerning the two systems of theology commented on so much by the learned everywhere, we came to Rush Medical College, one of the great centers of learning in the Northwest, there to pursue further our medical studies. At this place we were to be taught by you, gentlemen, who are thoroughly familiar with the structure and design of all the visible, or material portions of man; that part, upon the study of which alone, is founded modern theology; and having often heard it said that "learned" medical men were generally disbelievers in revelation, great was our desire to know what would be said by you, our newly chosen preceptors, upon questions of such vital importance.

The college term commenced—our desires were immediately gratified. Three of your number, including the professor of anatomy, in your introductory lectures, gave us to understand, and that very emphatically, that they do not regard men as baboons, nor the descendants of baboons. Neither do they accept the theory that man's mental manifestations are a species of magnetism; and much less still do they believe that in death man is annihilated.

Seven others of your number addressed us, all acquiescing in the views presented by the three I have mentioned. After this, several of your number were asked individually, outside of the lecture room, what their views were upon the three questions with which I commenced this discourse. Their replies were in perfect harmony with revelation.

Fifteen days passed. Students were arriving from all parts of our country. California and Southern Texas, the East and the distant North, were represented. Never before, so early in the term, had Rush such an assemblage of students within her walls. Never before had you, her professors, commenced a course of lectures with such prospects, such pleasure, such anticipations.

But now the fullness of time had arrived for Chicago, the wonder of the world, the pride of our nation, to, phœnix-like, renew her youth, and your stately edifice, which added so much to her grandeur and reputation, must necessarily be consumed in the general conflagration; that, purified and beautified, it might be the better fitted for its exalted position in the new and magnificent Chicago of the future.

We students, being uninformed in this peculiarity of your city, supposed a terrible and almost irreparable calamity had befallen the college, and mourned with heartfelt sorrow what we believed her tragic and untimely fate. But soon we were informed, though the building was consumed, the college still lived; and not only lived, but that its life was intensified. The professor, who, previous to the fire, must necessarily have three skeletons and two fresh subjects, in order to deliver a successful course of lectures, now affirmed, that with one bone and standing on the open prairie, he could deliver a better course of lectures on anatomy than he had ever before delivered at Rush Medical College.

On hearing this we rallied, and to our joy ascertained the fact, that what we had looked upon as Rush Medical College, was no more necessary to its existence than the aged body of Israel's great law-giver, slumbering in the plains of Moab, was to the existence of Moses, who on Hermon's top held communion with our Savior and Elijah.

*Rush truly lived.* Lectures commenced again in earnest, and have continued in earnest until the present time, the close of the term. As a *resume* of what each professor has said would be out of place here, owing to our limited time, we must content ourselves by saying, that during the term all obsolete theories, that serve only to confuse the student's mind, have been passed by unnoticed, and that the living, breathing, practical truths of the present alone have engaged your attention. The result has been, a telling effect upon the minds of the class.

So, gentlemen of the Faculty, taking the whole term of lectures, from its commencement to its conclusion, notwithstanding the "world's fire," we have no hesitancy in saying it has been a grand success, and that we believe no class, through the whole length and breadth of our country, have received so much practical knowledge as we have; and if our future as practitioners be not one of success, we shall charge the defect to our own natural inability, not to a lack of proper instruction.

And now before we separate, we promise an unswerving fidelity to the broad and liberal principles you have so earnestly and forcibly presented for our adoption; and that in the future when you see or hear from a member of the class of eighteen hundred and seventy-one and seventy-two, it matters not where he may be, treading the cold and frozen soil of the distant North, or scorched beneath the burning rays of a tropical sun—on the stormy coasts of the Atlantic, or basking in the more genial climes of the Pacific, you shall learn, true as the needle to the pole he remains to those great and unchanging principles of medicine he first learned here; and that with a bold front and a firm tread, he is marching on in the faithful discharge of his professional duties, bearing aloft unsullied the standard of Rush, and proudly claiming her as his *Alma Mater*.

One thing more before I close. Even here in Chicago we are asked if the college building is again to be reconstructed. When we reach our homes the same question will be asked us there. Our answer shall be, Yes, just as certain as the sun continues to rise and to shine. Rush is part of Chicago; their interests are one and inseparable. Earthquakes may level, tornadoes may scatter, waters may drown, or fires consume, but before the rumbling of the earthquake's tread dies on the ear, the air becomes calm from the tornado, the water dries in the streets, or the ashes become cool from the fire, Chicago will begin to rise again, and with it, Rush, and that to increased splendor and glory. Farewell!

Companions of the class of eighteen hundred and seventy-one and seventy-two, you have heard my promise. I shall not say to you, hold it inviolable; I know you will do this. The readiness with which you rallied to the standard of Rush immediately after the fire, the zeal and ability you have manifested ever since, convinces me you are fully alive to the responsibility of your position, and that as men you will unhesitatingly discharge them.

Good bye.

### PRESENTATION.

A pleasant episode occurred in the exercises. This was a presentation of a handsome case of surgical instruments to Dr. Gunn, Professor of Surgery. The presentation speech was made by Dr. A. N. Richardson, of LaMoille, Illinois, in behalf of the class. It was happily worded, and abounded in neat puns on the names of the faculty, which were received with applause and laughter.

Professor Gunn replied in an equally happy manner, thanking the class warmly for this token of their esteem, and his stars that had inspired them to bestow so appropriate a gift, and not a cane or a chair.

The concluding portion of the exercises was the following valedictory address, delivered by Jas. H. Etheridge, Professor of Materia Medica and Medical Jurisprudence:

### VALEDICTORY.

*Gentlemen :*

The present occasion is one fraught with great interest to you, because it records one of the few intensely interesting points in the career of a medical man. The hopes and aspirations filling your hearts this evening, would, if drawn upon canvass by a skillful artist, present one of the most curious and interesting pictures one can imagine. The past few years have been occupied with assiduous study. Earnestness has characterized your every effort. The sanctity of the dissecting and lecture rooms and clinical amphitheatres has not been violated by the spirit of trifling. Steady perseverance has conquered many a difficult point for you, and you feel proud of the ability to stand here this evening and demand at our hands the evidence of our confidence in you to assume the title of physicians and surgeons.

I think no medical class ever encountered greater inconveniences in their attendance on a final lecture course than the present one. Within ten days of the introductory lecture, our college, with all its means of illustration, was totally destroyed in the Great

Fire. Students were scattered in every direction, many of them losing their books and clothes, and some hardly escaping with their lives. In the great flood of charity and kind remembrances that came in upon us immediately after that memorable day, we were most touchingly remembered. Before our college ruins cooled we received letters and telegrams tendering us aid of every description. Other colleges kindly offered to take our students and instruct them gratis, turning them over to us for final examinations and graduation. One college offered to share lecture rooms with us, and one other college in an Atlantic city tendered us their whole building during the entire winter. Some physicians wrote us to draw on them at sight for money, and many letters from our Alumni anxiously inquired, What can we do for our *Alma Mater?* Nothing daunted by the immense loss sustained by the faculty in the destruction of our beautiful building, our first inquiry was, where can we best continue our winter's work? Then came these testimonies of anxiety about our condition, and these were a stimulus such as no sick man ever knew. The kindness of the County Hospital committee, which nothing can efface, allowed us to avail ourselves of their amphitheatre, and in less than five days from the fire our Professor of Anatomy reopened lectures, appearing to the few students present like the first *ray* of light after a dark, horrible night. From that time to the present, lectures have been uninterruptedly continued.

During the past session, no one has regretted so much as the faculty, the necessity of abandoning many contemplated experiments and illustrations, which could have been consummated only in the old amphitheatres where this course of lectures was commenced. Another very annoying circumstance has been the disadvantageous manner of studying practical anatomy. Through the generous courtesy of the faculty of the Chicago Medical College—the first to extend to us a helping hand—you have prosecuted your anatomical researches in their building.

Cheerfully have you met all these obstacles and overcome them, laboring day by day incessantly. At the end of last week—examination week—one of your number told me that, during that period he had passed an interval of sixty-five hours without sleep, resorting to caffeine and phosphoric acid to carry him through. Such dreadful anxiety—needless, to be sure—the public know but little of. Medical men appreciate it, however, and I can assure you that it is with peculiar pleasure that the faculty of this college confer degrees upon you this evening.

I have thought it best, gentlemen, to call your attention to a few points of interest, selected at random, knowing that at some future day your attention will be demanded by them, perhaps. I offer no apology for particularizing, because your student days have been occupied in acquiring rudiments and facts, positively known, and you have not had time to become familiar with speculative, undetermined matters in our profession, to any marked extent as yet.

It is well for us at times to pause and question, "Whither do our scientific investigations tend?" Do they corroborate, or are they subversive of, the cardinal points of Christian belief? We hear a great deal now-a-days about the conflict between science and religion. Only a few years ago geologists and theologians were considered hostile. The former advanced true yet startling statements, which the theologians considered blasphemous. Now these scholars embrace each other with sweet delight, and both find that bible doctrines are made immensely stronger by geological investigations.

The various sciences have from time to time promulgated ideas that were considered antagonistic to religion at the time, and profound agitation resulted in the minds of the devout. But farther investigation and more familiar acquaintance with new truths of science, have, without exception, managed, with satisfactory success, to place *religious* truths on an immovable foundation.

Lately physiological investigation has offered a morsel for the religious world to swallow which well nigh resulted in strangulation to the latter. What is known as the "Physical Basis of Life" doctrine was first advanced by Huxley, in Edinburgh, in 1868, in a lecture one Sunday evening, and that lecture has created an immense disturbance in the minds of scientific and religious people. Scientists are stirred up because many of them cannot agree with the learned lecturer. Many think that his conclusions are erroneous, and many others think he has made a great discovery. Theologians consider that the doctrine of "Physical Basis of Life" is totally at variance with the doctrines of revealed religion —incompatible with religious thought.

Huxley attempts to prove, to use his own words, "that there is some one kind of matter which is common to all living beings, and that their endless diversities are bound together by a physical as well as ideal unity." That there is a community of form and structure of the ultimate particle—that is, the cell—between a man and a dog—between a man and a thistle—between a man and a whale or fungus, or marine ascidian—"between the flower which a girl wears in her hair and the blood which courses through her youthful veins"—"between the dense and resisting mass of oak or the strong fabric of the tortoise, and those broad disks of glassy jelly which may be seen pulsating through the waters of a calm sea, but which drain away to mere films in the hand which raises them from their element." This peculiar matter "common to all living beings" he terms "protoplasm," and he tries very ingeniously to show that there is a threefold unity in all protoplasms—a unity of faculty, form and substance, and that, consequently, there is a common basis underlying all varieties of vital existence.

After having proven, as he considers, the identity in all living structures, of the particular substance in which life resides, he endeavors to tell *what life is.* He claims that life is not a mysterious something—like the soul or mind added to the body, as we have always been taught—but that it is a force correlative with other forces of nature; that for aught we know it may be only a modified form of heat or motion; that all living phenomena peculiar to living beings are only physical and chemical phenomena. He and his school claim that this force is reckoned with other forces. When it occurs in the *living* beings it is to be regarded as *vital force*—when it occurs elsewhere it is only a physical force.

This ingenious theory startled the world, and many scholarly gymnasts have promptly stepped forward into the arena of public debate, and arrayed themselves into hostile parties, and had several struggles over the matter, contesting solely on its scientific merits. Many of the clergy consider the "physical basis" doctrine directly antagonistic to the spirit of revealed religion. Even some of the scientific men drag in the consideration of religion in their opposition to Huxley. Beale says: "If it were true that the facts of science really taught that all phenomena peculiar to living beings were in reality only physical and chemical, the very ground out of which religious belief springs would be dissipated. For if I were sure that the formation of my body and the action of the living matter within me were certainly due to the properties of the particles of which my framework is constructed, how could I believe that I was nevertheless designed and created by the power and wisdom of God? If the physical views be accepted, surely the abandonment of a God, of Divinity of every kind, of immortality and free agency, is only a question of time. * * * * * If it is certain that, if the doctrines which have been lately so strongly advocated had been proved to rest upon a sure foundation, a complete and widespread revolution in religious belief would have occurred." Exactly why such a revolution would occur he does not state. He makes a great many such assertions as I have just quoted.

Again, he says: "If the formation and action of our tissues and organs are really due to the properties of the particles constituting the materials of our body, it is difficult to understand what influence a God could be supposed to exert after the particles had been created in the first beginning and had been endowed with their properties. Does not such doctrine, I would ask, strike at the root of the idea of a living God, and aim at accounting for all phenomena of this world by law, independent of will, law, or design? In such a scheme, neither a superintending Providence, nor a Personal God, nor Christianity, could have place."

Should you wish to pursue this subject farther, you will find it full of absorbing interest. The utter want of good foundation for Huxley's doctrine has been impressed on my mind thoroughly, and the same result may await you.

The Nervous System is a broad, comparatively uninvestigated field, that is just now demanding much attention. Pioneers are daily developing new facts concerning it, that explain many physical phenomena heretofore enveloped in mystery, and that elucidate the action of drugs on our systems. Difficulty of investigation of the duties and properties of any one portion of our systems, is perhaps nowhere so manifest as in the cerebro-spinal, and ganglionic systems. Their physiology and pathology are wrought out and made known only after slow, patient work of many years. The past decade has witnessed most important and rapid strides in the advance of our acquaintance with nervous phenomena, and many of the discoveries of this period tend to revolutionize and modify our notions of disease and treatment, and very many articles in our standard works on Theory and Practice must be re-written. A whole series of groundless doctrines about disease and its treatment have been put forward, based on some supposed perversion of the function of that part of the ganglionic system known as the vaso motor nerves. Still, notwithstanding the immense progress which has been made since 1860, our knowledge of these particular nerves is not yet exact enough to afford an altogether safe ground for pathological speculation. Many of the most striking phenomena of disease, particularly those of fever, inflammation and collapse, must find their explanation in disordered vascular innervation. We are now in a better position to understand these disorders, yet our knowledge is still insufficient, and their investigation is full of difficulties and perplexities.

The history of one pathological condition, inflammation, indicates the progress of therapeutics in a great measure. Formerly it was held that all diseases depended on inflammation—that there was no disease without inflammation. Then a common cold was simple inflammation ; nervous debility was inflammation ; hysteria the result of a hidden inflammation ; fevers were the purest inflammation. Even scarlet fever and measles were inflammation of the highest order. Now we regard this condition in most cases where it does occur, as a secondary consideration. Then this disorder was treated with the most heroic, wholesale, indiscriminate blood-letting, the inflamed part being considered too full of life, and the blood in it held there by hydrostatic principles. A friend of mine once informed me, that very many years ago, his old family physician, a man of irreproachable respectability, believed that there was only one remedy for measles, and that was bleeding. It was the rarest accident when one of his patients recovered. They died like flies in October, and, strangely enough, he profoundly believed that they died because he had not bled them early or copiously enough.

Dr. Laycock, of Edinburgh, in a recently published course of lectures on the nervous system and its influence on diseases of organs and tissues, advances some new ideas that promise to be of signal service in treating disease. According to his observations, " diseased states of organs or tissues are reversions or degenerations to a lower form of evolutions of tissues and organs." Thus, nervous debility, considered as a deficiency of trophic energy, will coincide with anatomical and chemical tissue changes of a lower type. The production of uric acid, the *materies morbi* of gout, is an illustration. It is a product of transformation of tissues of birds and reptiles; in man it is abnormal, being a retrocession from urea. So lactic acid appears to be the result of a retrocession in muscular transformation from a higher compound. Carbons and hydrocarbons, as pigments and fats, follow the same law as to place of production. He has also introduced into the study of medicine doctrines regarding diathetic diseases which differ widely from those current, in that they are founded on the evolution of tissues and organs, and possess, therefore, a solid basis in histological anatomy, instead of merely the natural history of disease. All the tissues of the body are evolved from the two divisions of the germinal membrane—the "mucous" and "serous" layers. From the serous layer are evolved the whole voluntary motor apparatus of bones, ligaments, aponeurosis, muscles and serous tissues. Consequently they are all related to each other by common origin, and, nutritionally and diathetically. From the mucous layer is evolved the nutritive apparatus. The circulatory system springs from both the serous and mucous layers. Keeping in mind the *origin* of the various tissues of the body, diathetic diseases and degenerations are easily comprehended. Especially easy is it to comprehend the hereditary tendencies to these diseases and degenerations. By a consideration of diathetic principles, medicines formerly administered with great doubt can now be given with more confidence, and disorders uncontrolable by ordinary routine treatment, more readily succumb to diathetic remedies. A knowledge of the particular diathesis of each patient, suggests to us at once the particular organs or tissues liable to be affected in each, and can be used as an accurate guide to sound and definite conclusions.

In the same manner does he classify skin diseases, basing his classification on the threefold nature of the tissues (the mucous, vascular, and fibrous or connective, including the muscular) which, each or all, may be affected.

Guided by the law of evolution, the same author works out a still more interesting point in therapeutics. Many years ago the late Dr. Turner, of the University of London, and Sir Robert Christison, made experiments on plants with opium and similar drugs. They showed that a community of influence of the drugs existed as to the tissues of plants and animals. According to Dr. Laycock's method, the motor vis nervosa is an evolution of the directive and "purposive" qualities as shown in plants as well as in animals—in short, in all living things. Hence, it follows that drugs believed to act exclusively on the nervous system, act also on the tissues. In this way is explained the action of strychnine in certain forms of emphysema with bronchitis; and colocynth, aloes, senna, wormwood and chamomile are referred to as drugs possessing a combined influence on the tissues and nervous system. It is obvious that such a principle must modify all our therapeutical views.

The phenomenon of reflex action is one of interest, and lately has proved to be the key to a very important discovery. The simplest illustration of it is, in tickling the bottom of the foot and observing the spasmodic jerking of the leg away from the object producing the tickling. Every physician knows the explanation of the jerking motion; and many, very many intelligent people who are not physiologists understand that reflex action is a system of telegraphing on the nerves, and explain the process thus: "The fingers tickled the foot and certain nerves conveyed that sensation to the spinal cord and brain, and the mind sent down a despatch along certain *other* nerves, which contracted the muscles and produced the jerking." To put it a little more learnedly, the vis nervosa, or nerve force, travels upwards on sensitive nerves, and downwards on motor nerves. Investigation proves that this force never travels in any other direction; and furthermore, that all growth, nutrition and development proceed in the same, and in no other direction; and still farther, that degeneration of nerves passes in precisely the same direction. Suppose a motor nerve be divided at the elbow, what results? Degeneration of that nerve along its course to its minutest termination down into the hand and fingers, and paralysis of the parts thus supplied. Hence, conversely, if we find paralysis of any particular part supplied by a certain nerve, we conclude that that nerve is out of order. Strabismus occasionally occurs in this way. Again: suppose we divide a *sensory* nerve at the elbow, what results? Degeneration of that nerve from the section up the arm into the brachial flexus—into the cord and along its individual filaments up the cord into the base of the brain. Not unfrequently, sensitive fibres run up into the hemispheres, the organ of thought and perception. If one of these fibres be destroyed, sometimes disease of the mind or insanity results. Hence it is we understand how some diseases result in broken minds—how injuries eventuate in insanity. I know a man now in an insane asylum for the fifth or sixth time, made crazy by the excessive use of tobacco. Undoubtedly the cause was the effect of smoking on the sensitive filaments of the pneumogastric. Laycock mentions a man who had his finger jammed in the door of a rail car, so that a portion was squeezed off. Now follow the symptoms as they developed, and see how the degeneration of the sensitive nerve proceeded. I quote the writer: "The finger healed; but in the course of a month after the injury, he had a slight tetanic symptom, and in a few days after that, a sort of a fit. He now also complained of numbness and strange sensations in his hand and arm, twitching of the face, and a sense of weariness and loss of strength, so that although previously in robust health, he was unable to undergo even slight exertion without a feeling of fatigue. He resumed his office work for six months, but slowly got worse, becoming highly nervous and dreadfully depressed. He next had numbness of the body, and faintness; then by degrees his powers of speech, of motion and vision failed, and at last he died, eighteen months after the accident."

We find attempts have been made to teach that there are distinct cerebro-spinal centres set aside for the regulation of distinct trophic areas. It is claimed, that for the maintenance of so conspicuous a harmony of function of the trophic system over an extensive and complex series of areas, there must be one centre, or a series of centres. Admitting this claim, renders easy the explanation of very many obscure diseases, and explodes many pet theories of diseases and symptoms.

In continuation of the discovery of the law of degeneration of nerve filaments, a discovery prompted by the phenomenon of reflex action, I wish to allude for a moment to a region of the cord, named by Waller and Budge the cilio-spinal tract. By experiments on this tract, certain results can be produced on the eye. It is situated in the neck. Claude Bernard describes a similar tract, but he embraces more area, and denominates it the "oculo-spinal" tract. Certain nerve filaments coming off with the second pair of dorsal nerves—the lower part of this oculo-spinal tract—undergo degeneration upon section, *from* the cord, that is, *centrifugally;* hence, the conclusion is, these filaments must be *motor* filaments, because motor nerve force, motor nerve growth and degeneration travel *from* the cord. Section of motor nerves means paralysis to some organ or part, whatever it may be; and upon cutting off these motor filaments of the second dorsal nerves, we naturally look out for this paralysis, and we find it in the eye—the iris being dilated. Hence dilation of the iris means paralysis. In those diseases where we have pupilary dilation and contraction we naturally conclude we have another point of explanation of the effects of disease on our systems. Cannot we obtain some clue to the effects of the solanaceæ, whose uniform effect on the eye is dilation of the pupil? Do we not find an important suggestion, too, as to the antagonistic effects of opium and belladonna? It is more easily comprehended how these remedies act in the delirium of fever and of tremens. Bernard has shown that the nerves coming from the last cervical and first and second dorsal vertebræ direct the innervation of the tissues of the eye upon which the protrusion of the eyeball, exophthalmos, one of the symptoms of heart disease, depends.

Laycock continues: "Now this region is a most important region clinically, both as to neurotic, functional and structural diseases of the heart, such as nervous palpitation, angina pectoris, and as to dropsies of the thorax, neck and arms."

The revelations of the spectroscope are astonishing, and it seems to me as though we are familiar with only one of a hundred, perhaps a thousand, invaluable helps it will some day yield to us. The one help I refer to, is, its detection of blood stains—an item often of thrilling interest to medical men and to society.

The instrument has been explained and exhibited to you by the Professor of Chemistry. The delicacy and certainty of its revelations most profoundly impress the merest tyro, and it seems to me that in medical experience will it prove of most important service. It demonstrates the presence of common salt in the atmosphere, for salt is present everywhere; it pervades the earth and air universally; it dances in the sunbeam; and we can by no means get rid of it, do what we will. We cannot leave a clean platinum wire exposed to air a moment without particles of sodium becoming attached to it. The most delicate chemical manipulations never have determined this fact, yet the spectroscope makes it clear. The smallest possible amount of any salt dropped into a tub of water cannot be detected by the most careful tests, yet the spectroscope quickly detects it. A physician, convinced that some cases of typhoid fever in one of his families were caused by drain poison getting into the well that supplied drinking water to the family, determined on testing the well water. Chemical tests revealed nothing wrong. The residue from evaporation of some of the water yielded no very positive result with the spectroscope. He then put some lithium into the house drain, and after several hours repeated his spectroscopic observation, and at once the brilliant, beautiful red line which lithium only produces, appeared. Plumbers soon found the defective sewer pipe, and no more typhoid has occurred in that house.

Astronomers have applied spectroscopic observations to the heavenly bodies, and the chemical nature, the physical constitution, and, within certain limits, the temperature and the density and velocity and direction, whether toward or from us, of many of the stars and planets, have been accurately ascertained. They announce that Saturn has an atmosphere similar to ours; so has Mars; that there is hydrogen in the sun, etc.

Microscopists are having stands constructed to which they attach spectroscopes, and apply observations to infinitely small objects. I fancy that in this way is this wonderful instrument to be of signal service to the medical man in carrying on minutest and most certain investigations in the products of disease, and in determining the exact nature of zymotic poisons, and settling the perplexing question of disease germs, beyond a doubt. A simple blood corpuscle, dried and shrunken, and having lost all semblance to a corpuscle, can scarcely be recognized after moistening, by the most powerful microscope, yet the spectroscope indicates it quickly and unmistakably. Spectra of the various products of our bodies are now being recorded by observers, and I imagine the time is not far distant when this instrument will afford as rich means of clinical investigation as the miscroscope or stethoscope. Possibly the time is not far distant when every physician will have a pocket spectroscope, that he can devote to clinical purposes, and tell precisely the nature of the action, whether deficient or too abundant, of the skin, kidneys, lungs, liver, and every other organ of the body. This instrument will, some day, be equally esteemed with the stethoscope and sphygmograph, and when these three go hand in hand with the physician in his investigations, clinically, we will doubtless hear of some enterprising Doctor applying the auto-phoroscope to sounds derived from the various forms of thoracic diseases, and to those of intestinal gurgling in bowel disorders, obtaining tracings as accurate and reliable as the sphygmograph yields, and upon them found some new and startling disease theory.

In the great work which lies before you, gentlemen, we bid you go forth full of bright hopes, and strong in your determination to overcome difficulties by patience, courage and perseverance. But remember always that the one object of your lives should be to seek after and maintain truth. Be true to yourselves, and you will be true to your professional calling, than which there is nothing higher and grander in this life. Truth should penetrate every act of your professional, as of your private life. It will make you careful in diagnosis and sound in therapeutics, because it will enable you to sift the apparent from the real—the false from the true. It will help you to weigh symptoms, to estimate them at their true value, and by knowing accurately, that is, truthfully, the inward conditions, of which symptoms are but the outward expression, you will be able, with full confidence, to apply the true remedy.

But success of this kind is only obtainable by the persistent practice of truth in every minute particular of your daily life. You cannot play fast and loose with truth and falsehood, for one or the other will, in the end, be sure to prevail : and remember that "falsehood is never so successful as when she baits her hook with truth ; and that no opinions so fatally mislead as those that are not wholly wrong, just as no watches so effectually deceive the wearer as those that are sometimes right."

After the close of the Valedictory the Benediction was pronounced by the Rev. Chaplain, and the assemblage dispersed.

